# Hydroxymethylation and Metals: A Potential Epigenetic Marker for Effects of Toxic Exposures

**DOI:** 10.1289/ehp.122-A251

**Published:** 2014-09-01

**Authors:** Lindsey Konkel

**Affiliations:** Lindsey Konkel is a Worcester, MA–based journalist who reports on science, health, and the environment. She is an editor for *Environmental Health News* and *The Daily Climate*.

It isn’t well understood how chronic low-level exposures to toxic metals contribute to disease, but a growing number of studies suggest epigenetic mechanisms may play a role.^1^^,^^2^^,^^3^^,^^4^ In this issue of *EHP*, investigators report a novel association between metals exposure and DNA hydroxymethylation, an epigenetic modification that has only recently entered the research spotlight.^5^

Epigenetic modifications can influence gene expression without changing the genome sequence. DNA methylation—the addition of a methyl group, typically to a CpG site (where a cytosine base is followed by a guanine base)—is an important step for a number of cell processes, including embryonic development and maintenance of chromosomal stability.^6^ Aberrant levels of DNA methylation have been reported in association with a number of diseases.^7^^,^^8^

**Figure:**
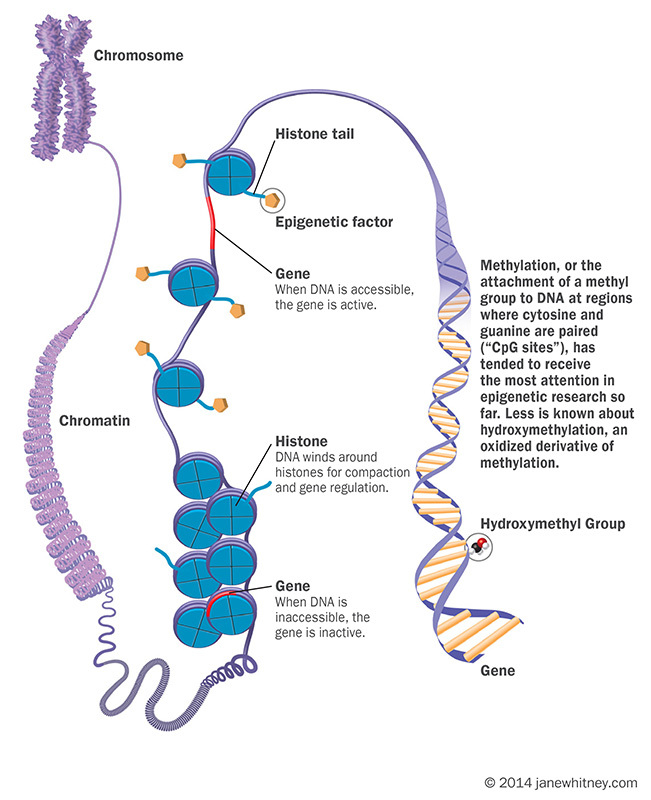
Methylation, or the attachment of a methyl group to DNA at regions where cytosine and guanine are paired (“CpG sites”), has tended to receive the most attention in epigenetic research so far. Less is known about hydroxymethylation, an oxidized derivative of methylation. © 2014 janewhitney.com

Less is known about DNA hydroxymethylation—an oxidized derivative of DNA methylation. Recent studies suggest it, like DNA methylation, can regulate the expression of genes involved in certain cancers, and that it also may modulate methylation levels.^9^

The current study was led by Maria Tellez-Plaza, now an epidemiologist at the Institute for Biomedical Research at Hospital Clinic of Valencia. She and her colleagues used blood and urine samples collected from 48 American Indian men and women who participated in the Strong Heart Study.^10^^,^^11^ Sets of samples were collected during two clinic visits, the first between 1989 and 1991 and the second between 1997 and 1999. The researchers looked at genomewide DNA methylation and hydroxymethylation in participants’ blood as well as levels of arsenic and cadmium in their urine.

Participants with the highest cadmium exposures had significantly higher blood levels of the DNA methylation marker 5-methylcytosine than people with lower cadmium measurements. Those with a faster arsenic metabolism profile in their urine had significantly higher levels of the hydroxymethylation marker 5-hydroxymethylcytosine. These associations remained after the researchers adjusted for other factors associated with DNA methylation, such as age, sex, body mass, and smoking status. The associations also remained consistent between the two clinic visits, several years apart.^5^

Cadmium concentrations in the study participants were higher than levels recorded in the general U.S. population over the same time period, says Tellez-Plaza. Cadmium exposure can come from cigarette smoke, from certain industrial emissions (e.g., smelters, incinerators, and power plants), and from some paints, glazes, batteries, and electronics.^12^ Arsenic levels were moderate to high compared with the general population. Tellez-Plaza says this was especially true of participants from Arizona and the Dakotas, where arsenic in drinking water commonly exceeds the current U.S. Environmental Protection Agency maximum contaminant level of 10 µg/L.^13^

The researchers also reported a positive association between DNA methylation and hydroxymethylation levels.^5^ This novel finding “suggests environmental determinants may be common,” Tellez-Plaza says.

“The study shows that methylation and hydroxymethylation are associated with these environmental metals. Further research is needed to explain the association between metal exposure, these epigenetic marks, and various health effects,” says Rebecca Fry, a molecular toxicologist at the University of North Carolina School of Public Health. Fry was not involved in the present study.

In order to understand the potential role played by environmental exposures and associated epigenetic modifications in disease processes, it will be critical to understand their functional effects on gene and protein expression, Fry says. “This study paves the way for subsequent research that could examine these biomarkers in the context of gene-specific and functional changes,” she says.

Next, Tellez-Plaza and colleagues hope to confirm their preliminary findings in a larger study population. This could help clarify if one measure or the other—DNA methylation or hydroxymethylation—is preferable for detecting epigenetic effects.
